# Wellness Influencer Responses to COVID-19 Vaccines on Social Media: A Longitudinal Observational Study

**DOI:** 10.2196/56651

**Published:** 2024-11-27

**Authors:** Gabrielle O'Brien, Ronith Ganjigunta, Paramveer S Dhillon

**Affiliations:** 1 School of Information University of Michigan Ann Arbor, MI United States; 2 Department of Electrical Engineering and Computer Science University of Michigan Ann Arbor, MI United States

**Keywords:** social media, COVID-19, vaccination, personal brands, public health, wellness, global health, pandemic, Twitter, tweets, vaccine, longitudinal design, wellness influencers, hand-annotation, anti-vaccination, infodemiology

## Abstract

**Background:**

Online wellness influencers (individuals dispensing unregulated health and wellness advice over social media) may have incentives to oppose traditional medical authorities. Their messaging may decrease the overall effectiveness of public health campaigns during global health crises like the COVID-19 pandemic.

**Objective:**

This study aimed to probe how wellness influencers respond to a public health campaign; we examined how a sample of wellness influencers on Twitter (rebranded as X in 2023) identified before the COVID-19 pandemic on Twitter took stances on the COVID-19 vaccine during 2020-2022. We evaluated the prevalence of provaccination messaging among wellness influencers compared with a control group, as well as the rhetorical strategies these influencers used when supporting or opposing vaccination.

**Methods:**

Following a longitudinal design, wellness influencer accounts were identified on Twitter from a random sample of tweets posted in 2019. Accounts were identified using a combination of topic modeling and hand-annotation for adherence to influencer criteria. Their tweets from 2020-2022 containing vaccine keywords were collected and labeled as pro- or antivaccination stances using a language model. We compared their stances to a control group of noninfluencer accounts that discussed similar health topics before the pandemic using a generalized linear model with mixed effects and a nearest-neighbors classifier. We also used topic modeling to locate key themes in influencer’s pro- and antivaccine messages.

**Results:**

Wellness influencers (n=161) had lower rates of provaccination stances in their on-topic tweets (20%, 614/3045) compared with controls (n=242 accounts, with 42% or 3201/7584 provaccination tweets). Using a generalized linear model of tweet stance with mixed effects to model tweets from the same account, the main effect of the group was significant (β_1_=–2.2668, SE=0*.*2940; *P*<*.*001). Covariate analysis suggests an association between antivaccination tweets and accounts representing individuals (β=–0*.*9591, SE=0*.*2917; *P*=*.*001) but not social network position. A complementary modeling exercise of stance within user accounts showed a significant difference in the proportion of antivaccination users by group (*χ*^2^_1_[N=321]=36*.*1, *P<.*001). While nearly half of the influencer accounts were labeled by a K-nearest neighbor classifier as predominantly antivaccination (48%, 58/120), only 16% of control accounts were labeled this way (33/201). Topic modeling of influencer tweets showed that the most prevalent antivaccination themes were protecting children, guarding against government overreach, and the corruption of the pharmaceutical industry. Provaccination messaging tended to encourage followers to take action or emphasize the efficacy of the vaccine.

**Conclusions:**

Wellness influencers showed higher rates of vaccine opposition compared with other accounts that participated in health discourse before the pandemic. This pattern supports the theory that unregulated wellness influencers have incentives to resist messaging from establishment authorities such as public health agencies.

## Introduction

Social media has profoundly transformed the landscape of health information sharing and dissemination. Platforms such as Facebook, Twitter (rebranded as X in 2023), and Reddit have become crucial venues for health discussions [1-6], where individuals navigating medical decision-making may find knowledge and community support [7-14]. At the same time, media platforms may provide opportunities for unverified and potentially dangerous medical misinformation to reach wider audiences [15-22].

Social media platforms democratize who gets to command an online audience, which presents both opportunities and liabilities for public understanding of science and medicine [23]. Scholars have noted the rise of a “wellness influencer” class, online personalities who dispense health and lifestyle advice as part of their personal brand [24-28]. For these influencers, a lack of professional credentials (such as medical or scientific training) is often a source of authority: by eschewing ties to traditional institutions like universities or public health agencies, influencers can position themselves as relatable, authentic, and uncorrupted. This rhetorical strategy may be especially effective when trust in longstanding institutions is low [24].

Indeed, there is converging evidence that in some countries (such as the United States), trust in science has diverged from trust in scientific institutions. Surveys indicate that while confidence in “science” remains strong, confidence in the professional and governmental organizations that traditionally represent scientific and medical authority has waned [29-31]. This has prompted sociological examinations of who gets to “speak for science” [27].

If social media influencers rush to fill the void left by skeptical attitudes toward conventional institutions, this may have important ramifications for public science communication strategies. While considerable focus has been directed toward online misinformation research in recent years, there are still important gaps in the literature. For example, while there are many case studies of wellness influencers contradicting the guidelines of medical and public health authorities [26,32], it is more difficult to measure the prevalence and persuasiveness of this content [33,34]. On the other hand, while there are numerous studies leveraging computational social science techniques to detect patterns in health information sharing in large volumes of social media data [3,35-41], it is still challenging to connect this work to sociological theories of who claims to speak authoritatively on health and why.

While it has been reported that a handful of influential Twitter accounts (some that could be reasonably described as wellness influencers) shared a disproportionate volume of antivaccine messages [42] during the pandemic, this type of measure does not directly establish the prevalence of those attitudes among wellness influencers generally. It remains possible that most wellness influencers did not oppose vaccination, and their salience in misinformation research is due to a small but active minority. If this is true, we will need to refine theories of how the authority of science is negotiated online.

In this study, we aim to directly test whether being a wellness influencer online before a major public health crisis is associated with sharing anti-establishment opinions toward a public health intervention later [43]. Specifically, we use the COVID-19 pandemic and the ensuing vaccine rollout as a probe, as there is a sizable foundation of research tools available for analyzing this type of social media content—particularly on Twitter, where we locate our study [44-47]. In addition to the researcher tools available for studying antivaccine attitudes on social media, we leverage a variety of methods from natural language processing, a family of techniques for analyzing unstructured text, that has been previously applied to understanding online social interactions around health matters ranging from mental illness [6,8,12,37,48,49] to nutrition [5,43,50].

If being a wellness influencer truly requires opposing traditional health authorities as a core part of brand building, then we should expect influencers who were established before the pandemic to voice antagonistic opinions toward vaccines (which are necessarily created, tested, and distributed by scientific and medical institutions). Our study uses a longitudinal design to test the hypothesis that wellness influencers active before the pandemic were more likely to express antivaccination attitudes during the pandemic compared with a control group of accounts. We use a previously developed language model [51], trained on a high-quality dataset of labeled tweets, to classify opinions expressed toward or against the COVID-19 vaccine in thousands of tweets collected throughout the pandemic.

Notably, we hand-annotate user accounts to establish a representative “wellness influencer” sample, operationalized here as individuals who provide health and wellness advice without formal regulatory constraints. In addition to investigating the prevalence of anti-public health attitudes among our sample of influencers, we also analyze the key themes and rhetorical tactics used by wellness influencers. This combination of quantitative and qualitative analyses provides a new estimate for the prevalence of anti-establishment health beliefs among a clearly defined segment of users, as well as insight into their messaging strategies.

## Methods

### Overview

**Figure 1:**
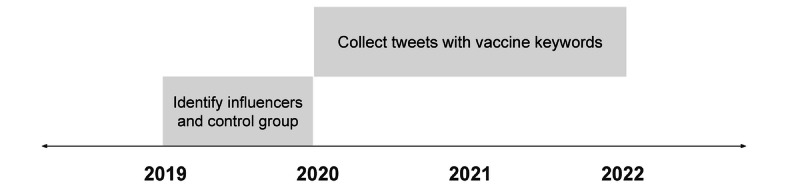
Diagram of the study design.

In our study, we first created a cohort of wellness influencers on Twitter (note that Twitter has been rebranded as X, but throughout the manuscript, we will refer to the platform as Twitter, as this was the name at the time of data collection). The cohort of influencers was selected according to definitions established by Baker and Rojek [25]. Specifically, these individuals are characterized by their online behavior providing health and wellness guidance directly to their followers. Subsequently, we tracked these influencers’ posts and perspectives on COVID-19 vaccination from the onset of the pandemic in 2020 until the end of 2022. This strategy is depicted in Figure 1. The source of our data was the Twitter Decahose, a 10% random sample of all tweets. Our analysis was restricted to English-language tweets only.

### Identifying Influencer Accounts Before the Pandemic

Our process for identifying wellness influencers began by analyzing Twitter activity in 2019 (a year before the COVID-19 outbreak), focusing on accounts that significantly contributed to health and wellness conversations, as evidenced by high retweet counts.

#### Estimating the 2019 Retweet Network for Health Discourse

To construct a retweet network on the topics of health and wellness, we began with the task of identifying a keyword list. Using all available 2019 tweets in the Decahose, we trained a word embedding model [52] using the Python Word2Vec library [53]. We then identified the nearest 400 tokens in the embedding space to the seed “#wellness.” We also identified the nearest 400 tokens to the nonhashtag form of the seed, “wellness.” This gave us 400 hashtags and 400 nonhashtag keywords. Using our keyword list, we looked for accounts that had used keywords at least 50 times in the 2019 tweet corpus. Note that the threshold of 50 is somewhat arbitrary and conservative; by choosing a relatively high threshold, we are selecting for accounts with a markedly high volume of tweets with health keywords, but this may exclude accounts with clear health opinions that post less frequently. This stage yielded a sample of 2414 accounts.

#### Clustering and Annotating Influencer Accounts

To simplify labeling the 2414 accounts as influencers or not, we first used an unsupervised learning approach to cluster the accounts based on their 2019 tweets. After identifying relevant clusters, we hand-annotated the users in those clusters.

Clustering was done with the BERTopic library [54], treating each user’s collected tweets as a single document. BERTopic uses a general-purpose, pretrained sentence embedding model [55] to map documents into a 768-dimensional space. Then, BERTopic applies a dimensionality reduction technique (Uniform Manifold Approximation and Projection [UMAP] [56]) and clusters the reduced embeddings using a hierarchical density-based clustering technique, hierarchical density-based spatial clustering of applications with noise **(**HDBSCAN) [57]. HDBSCAN automatically selects a parsimonious number of clusters (in this case, 36). All users were assigned to their closest cluster. We use BERTopic’s default models and hyperparameters for document embedding, dimensionality reduction, and clustering.

After documents have been clustered, BERTopic optionally allows users to fine-tune the keywords that are used to represent each cluster. This does not change the documents assigned to each cluster but may be useful to help researchers interpret the clusters. We used the KeyBert topic representation sub-model, as this approach has been suggested by the BERTopic authors to produce more interpretable topic clusters [54].

Following the manual inspection of the user clusters, exemplar tweets, and cluster keywords identified by BERTopic, we determined that 9 clusters dealt with wellness topics (other notable clusters represented news, politics, medical practice, and technology). There were more than 900 accounts in the selected clusters. We manually inspected these accounts for adherence to two criteria:

1. Individual accounts: The account needed to be that of an individual, such as a personal trainer, nutrition coach, book author, or entertainer, rather than an organization or company.
2. Wellness advice provision: The account was required to actively offer health and wellness tips to its followers, such as practical lifestyle suggestions or personal health strategies.

To judge if an account represented an individual, we supplemented the analysis of collected tweets with a manual scrape of Twitter bios and a collection of Twitter bios we had pulled from the API (application performing interface) before it became inaccessible (Twitter removed its free API, along with support for researcher API access, in February 2023; the Twitter API is required to programmatically access user bios, locations, follower and following counts, and full tweet history). Through this manual annotation process, we pinpointed 186 accounts that satisfied both prerequisites. A random sample of 10 such accounts, with example wellness advice, is presented in Table 1.

**Table 1:** Anonymized sample of 10 randomly selected accounts in the influencer group. Their Twitter bios have been paraphrased, and advice tweets have been lightly reworded to prevent reidentification.

Self-description from bio	Example advice
Spiritual guide	Add clay to smoothies to eliminate toxins from your body.
Tech executive	Drink 3 liters of water daily for a month for clearer skin.
Food blogger	Eat coconut flour for healthy digestion.
Lifestyle medicine consultant	Intermittent fasting will lower your cholesterol.
Alchemist and herbalist	For optimal health, have alkaline foods and high pH water.
Metabolic health coach	Be mindful about cutting carbohydrates from your diet for improved health.
Psychic	Improve brain health with nutritional supplement drinks.
Motivational coach	To relieve your anxiety, meditate.
Writer	Avoid junk food, as it cannot be digested by your body.
Pop culture fan	The best thing for your body and mind is a simple lifestyle.

#### Snowball Sampling

To increase our sample size, we also conducted a “snowball” sampling stage: using our curated list of wellness influencers, we identified any accounts they retweeted more than once in 2019 that also contained wellness keywords. We then manually annotated those accounts to ensure adherence to our influencer criteria, which expanded our influencer count to 264.

While snowball sampling is a commonplace strategy to expand a sample, it may exacerbate sampling biases in some ways. For example, wellness influencers may be especially likely to follow other influencers who share their demographic characteristics, topical interests, and beliefs, a well-known phenomenon called homophily [58,59]. Therefore, we caution that this second stage of sampling may increase sample size without necessarily improving our sample’s representativeness of the broader population of wellness influencer accounts.

#### Considering Twitter Bots

It is well-established that Twitter contains “bots,” automated accounts that may serve functions such as aggregating content, tweeting advertisements, or providing followers to paying clients [60]. Defining and measuring the prevalence of bot accounts is complex, as estimates can vary based on the measurement technique. However, one estimate from 2017 put the percentage between 9% and 15% [61], and another using Twitter data from 2022 estimated between 8% and 20%, depending on how inactive and deleted accounts were handled in the calculation [62].

Ideally, we would have liked to use a research tool like Botometer [63], a model for labeling probable bot accounts. However, due to the shuttering of the Twitter developer API to researchers, we were unable to obtain the API access required to use Botometer or similar services. Still, we can make some inferences about the role of bots in our sample. As our influencer selection process involved hand-labeling accounts that represent themselves as individuals, which relied on visual inspection of the accounts’ bios (when available) and tweets, this group does not contain any accounts that appeared to be obviously bots, such as content aggregators or accounts that only post e-commerce links.

It is possible—and in the case of high-profile public figures, perhaps likely—that some influencer accounts use social media management software to schedule tweets with links to their blogs and websites. However, we consider this sort of messaging to be in the “voice” of the individual’s personal brand and do not regard it as inherently problematic for identifying the individual’s vaccination stance.

### Analyzing Pandemic-Era Tweets From Influencers

#### Collecting Vaccine-Related Tweets

**Figure 2:**
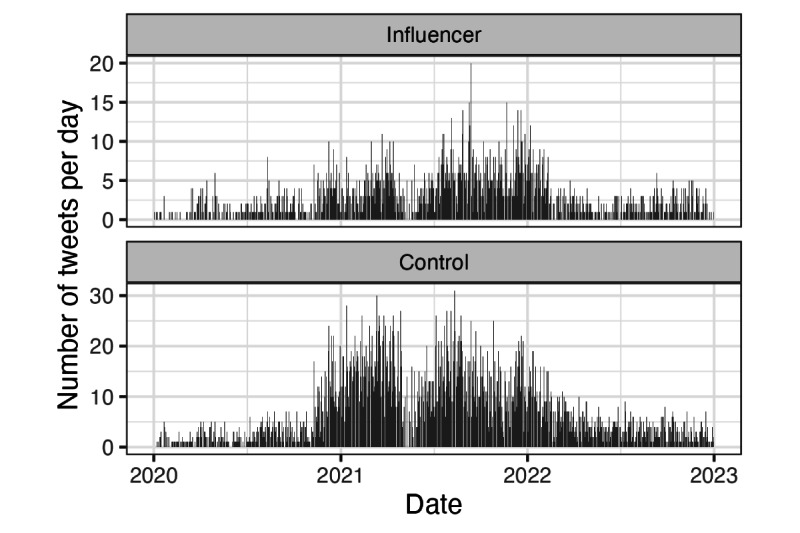
The number of tweets containing a vaccine-related keyword per day for each group over the investigation period.

We extracted all tweets between January 1, 2020, and December 31, 2022, from the Decahose for our list of influencers. Retweets made by influencers were retained, as these provide potentially important information about an account holder’s stance toward vaccination. We then filtered the collected tweets to only those containing a list of vaccine-related keywords and phrases. Our vaccine keyword list was created by combining lists from previously published studies of vaccine-related tweets [45,51]. Tweets were deduplicated before analysis, as some accounts reshared content on multiple days. The distribution of these tweets across the timeline is depicted in Figure 2.

#### Tweet Stance Labeling With the VaxxHesitancy Model

To evaluate the stance expressed in these tweets regarding the COVID-19 vaccination, we used a pretrained transformer-based model. This model was fine-tuned on the VaxxHesitancy dataset—a curated, annotated collection of 3101 English-language tweets about COVID-19 vaccines, gathered from November 2020 to April 2022 [51]. The tweets in this dataset were categorized by human annotators into one of four categories reflecting their stance:

1. Provaccination: Posts supportive of COVID-19 vaccination.
2. Antivaccination: Posts opposing COVID-19 vaccination and seeking to convince others to do the same.
3. Hesitant: Posts expressing uncertainty or a wish to delay or refuse vaccination.
4. Irrelevant: Posts not explicitly stating a stance on COVID-19 vaccination.

The published VaxxHesitancy dataset does not include the text content of the collected tweets, as it was published with the expectation that the Twitter API would remain freely available to researchers to reconstruct the tweet text from a list of IDs. After the Twitter API changes, it was no longer possible for us to reconstruct the tweet text from the ID list. In response to this challenge, the authors of the VaxxHesitancy dataset graciously shared with us a sequence classification model binary trained on a test set of 2670 tweets and evaluated on a test set of the remaining 431 (the test set consisted of only tweets that were at least double annotated with interrater agreement, ensuring high confidence in their stance labels). The stance classification model is based on the VaxxBert transformer model, with fine-tuning for the stance labeling task. The VaxxHesitancy team benchmarked the model with an accuracy of 74.5% and an *F*_1_-score of 70.5 on the test set for the 4-label classification task.

A preliminary analysis of our dataset revealed a notable pattern: tweets categorized as “hesitant” were usually from influencers who also produced “antivaccination” content. Despite these stances representing distinct categories at the tweet level, their frequent overlap within the same accounts indicated a shared behavioral pattern. Given that our original hypothesis did not distinguish between hesitant and antivaccination stances, and considering their common co-occurrence within the same users, we merged these categories for our analysis.

#### Tweet Topic Modeling

In our exploratory analysis of themes in influencers’ tweets, we again used the BERTopic library [54]. We separated tweets into two corpora: provaccination tweets by influencers and opposed tweets (comprised of hesitant and antivaccination tweets). For each corpus, we fit a BERTopic model (again, with the KeyBert topic representation submodel). Each tweet was treated as its own document for clustering.

#### Constructing A Control Group

Understanding the influence of wellness accounts on vaccination stances necessitates a benchmark for comparative analysis. Thus, we established a control group consisting of Twitter accounts that fall within similar topic domains as the influencer cohort but do not fulfill the criteria to be classified as influencers—those accounts either do not personify individuals or do not dispense wellness advice in the tweets we analyzed. Examples of such accounts include medical professionals and scientists who refrain from giving health advice on Twitter, public health campaign initiatives, various media organizations that feature wellness segments, advocates for mental health awareness, and commentators on public health. This control group will help us delineate the specific impact of wellness influencers as compared to the broader wellness discourse on the platform.

### Ethical Considerations for Studying Twitter Users

While some accounts in our sample correspond to public figures, others are unlikely to be widely known offline (for example, an account with followers numbering in the low thousands). Because we are studying individuals who take stances on a highly contentious public health issue, we expect these account holders to be at risk of harassment or other negative outcomes if they are identified in our reporting. Therefore, following the guidelines for social media researchers put forth by a committee at the Economic and Social Research Council at the University of Aberdeen [64], we present only anonymized quotes and account descriptions throughout our results.

## Results

### Influencer Stances Toward Vaccination

We began our analysis by examining overall patterns in vaccine-related tweets collected during the pandemic period (Table 2). Within the influencer group, we recovered 3045 relevant tweets from 161 accounts. Using the vaccine stance detection model, roughly 40% of these tweets were classified as containing no stance, roughly 20% were provaccination, and the remaining (~40%) tweets were labeled as hesitant or antivaccination. Meanwhile, for the control group, 7584 relevant tweets were collected from 242 accounts. The majority were labeled as not containing a stance (~50%) or provaccination (~40%), with only ~10% of tweets labeled hesitant or antivaccination. Overall, the proportion of stance-taking tweets was significantly higher in the influencer group (2-sample test for equality of proportions (*χ*^2^_1_[N=5616]=26*.*9, *P<.*001).

**Table 2:** Descriptive statistics of collected pandemic-era tweets.

		Influencers (n=161)		Control (n=242)	
Tweets with vaccine keywords, n		3045		7584	
**Stance detected, n**					
	No stance		1315		3698
	Provaccination		614		3201
	Hesitant or antivaccination		1116		685

Since our study focuses on evaluating attitudes toward the vaccination public health initiative, specifically measuring the percentage of provaccination tweets among those that expressed a clear stance, thus, we excluded tweets categorized as “No stance.” To account for the possibility of users contributing multiple tweets, we used a mixed-effects modeling approach to test the hypothesis that tweets from influencer accounts displayed more negative stances compared with other stance-expressive tweets.

We estimated a model, as outlined in Equation 1, with the stance of the tweet (coded as 1 for provaccination and 0 for antivaccination or hesitant) serving as the dependent variable. Our model incorporates random effects for users (α*_j_*) since a user could potentially contribute multiple tweets and an intercept term (β_0_). The coefficient of interest is β_1,_ which measures the impact of a user group (influencer vs. control) on tweet stance. Finally, given that the outcome variable (stance) was binary, our analysis was performed using a binomial generalized linear model to provide the most accurate representation of the data.

*s_ij_*=β_0_+α*_j_*+β_1_Group*_j_*+*ε_ij_*  **(1)**

where *i* is a tweet index, *j* is a user index, and tweet stance *s_ij_* is defined as

1*,* if tweet stance is labeled provaccination*,* or

0*,* if tweet stance is labeled negative or hesitant*.*

The model estimation results are presented in Table 3, where the control group is dummy-coded as the reference or baseline condition. The main effect of the group was significant (β_1_=–2.2668, SE=0*.*2940, *P*<*.*001). Based on the direction of the effect, tweets from the influencer group that express a stance on vaccination are, on average, more negative than tweets from the control group. We, therefore, reject the null hypothesis that stance-taking tweets are equally provaccination across groups.

To unpack the factors that may predispose an account to support or oppose vaccination, we also investigated the potential contributions of two other variables: “whether a Twitter account represents an individual and the network centrality of the account before the pandemic.” Accounts representing individuals may present opinions that are less moderated than accounts representing organizations or collectives, which could increase the propensity toward antivaccination messages. We did not have a strong a priori hypothesis about how an account’s position in the social graph would relate to vaccine stance; thus, this analysis should be considered post hoc.

**Table 3:** Mixed-effects generalized linear model of tweet stance.

	Dependent variable
	(Tweet stance, *s*_*ij*_)
Group	–2.2668 (0.2940)
Constant	2.1706 (0.1567)
Observations	5616
Log Likelihood	–1955.119
Akaike Information Criterion	3920.237
Bayesian Information Criterion	3953.404

#### Are Twitter Accounts Representing Individuals More Likely to Oppose Vaccination?

By definition, every influencer account must represent an individual. However, in the control group, 79 accounts were labeled as individuals (there were 29 accounts in the control group that we could not confidently label as individuals or not; these accounts are excluded from the following analysis). To test whether individual status adds explanatory value to a model of tweet stance, we added an additional term representing individual status to our baseline model:

*s_ij_*=β_0_+α*_j_*+β_1_Group*_j_*+β_2_Individual*_j_*+*ε_ij_*  **(2)**

where the indicator variable Individual is 1 if the account corresponds to an individual and 0 if not. Once again, as in Equation 2, the model fits coefficients αj (a random effect of the user), β0 (an intercept), and β1 (the effect of the user group). The new coefficient β2 refers to the main effect of individual status.

As can be seen from Table 4 (“Model 1”), the main effect of the group was still significant (β_1_=–1*.*3032, SE=0*.*3612; *P*<*.*001), and so was the main effect of individual status (β=–0*.*9591, SE=0*.*2917; *P*=*.*001). Based on the sign of the individual status main effect, we can see that tweets from accounts representing individuals were more likely to take antivaccination stances. Thus, we can see that the “individual” status helps explain additional variation in Tweet stance beyond what is explained by influencer status alone.

**Table 4:** Mixed-effects generalized linear models of tweet stance with covariates.

	Model 1	Model 2
Group	–1.3032 (0.3612)	–2.035 (0.282)
Individual	–0.9591 (0.2917)	—^a^
Log-centrality	—	–0.076 (0.095)
Constant	2.5420 (0.1965)	1.436 (1.002)
Observations	5206	5206
Log Likelihood	–1797.0530	–1803.645
Akaike Information Criterion	3606.1060	3615.289
Bayesian Information Criterion	3656.4510	3651.520

^a^Not available.

#### Does Social Network Position Affect Vaccine Stance?

Engagement levels on Twitter vary significantly from one account to another. Some enjoy high retweet rates, signaling frequent engagement, while others remain largely overlooked. Despite our selection of accounts based on their high retweet count, this does not preclude significant variability within our sample. To quantify engagement within health-related conversations on Twitter, we analyzed the in-degree centrality of each account using the retweet network (before COVID-19 in 2019).

In-degree centrality gauges an account’s influence by the volume of retweets it receives from distinct users. Upon comparison, we found no significant difference in the centrality of influencer and control group accounts (Wilcoxon rank-sum test; *W*=8401*.*5, *P*=*.*27). This indicates that being an influencer does not necessarily correlate with higher retweet centrality within our study’s context. We also tested a mixed-effects generalized linear model as before, except with each user’s centrality measure included as a main effect. Centrality was log-transformed before modeling, as it is nonnormally distributed with a long tail. The model results are presented in Table 4 (“Model 2”).

*s_ij_*=β_0_+α*_j_*+β_1_Group*_j_*+β_2_log-centrality*_j_*+*ε_ij_*  **(3)**

The main effect of log-centrality was not significant (β_2_=–0*.*076, SE=0*.*09472; *P*=*.*42). Hence, we did not find evidence that the connectedness of Twitter accounts prepandemic had an association with their stances on vaccinations during the pandemic.

### Cluster Analysis of Pro- and Antivaccination Accounts

So far, we have used a supervised learning-based modeling strategy to explore the influence of Twitter account properties on Tweet stance. An alternative analysis strategy is to model users’ stances in an unsupervised fashion, which yields more readily interpretable estimates of how many accounts in the influencer and control groups supported vaccination, respectively. To this end, we clustered users into two broad categories: vaccination supporters and opponents.

For all accounts that had at least one tweet labeled as containing a stance (120 influencer accounts and 201 control accounts), we calculated a stance vector as follows:



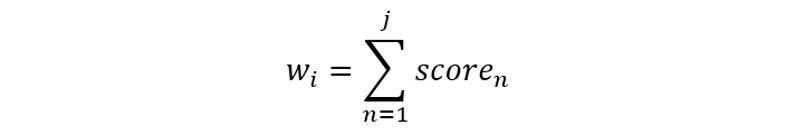

**(4)**


Where *i* is an index for each of the three stance labels produced by the VaxxHesitancy model (“positive,” “hesitant,” or “opposed”), n indexes every tweet with label *i* for a given account, and the “score” is the stance model’s confidence score for that label (a number between 0 and 1). The stance vector is normalized by dividing by the total number of tweets per account so that its values sum to 1.

For example, an account with two tweets labeled “opposed,” each with a confidence score of 1, would have a stance vector of [0, 0, 1]. If the account had two tweets, one labeled “opposed” and one “hesitant” (again with a confidence score of 1), the resulting vector would be [0, 0.5, 0.5]. Confidence scores are incorporated so that Tweets that are not confidently labeled by the model will contribute less to the stance vector.

Next, we used the *k*-means clustering algorithm to assign every account’s stance vector to *k*=2 clusters. Accounts from both groups (influencer and control) were pooled together for clustering. We did not attempt to correct the class imbalance (ie, that there were more control accounts than influencer accounts).

**Figure 3:**
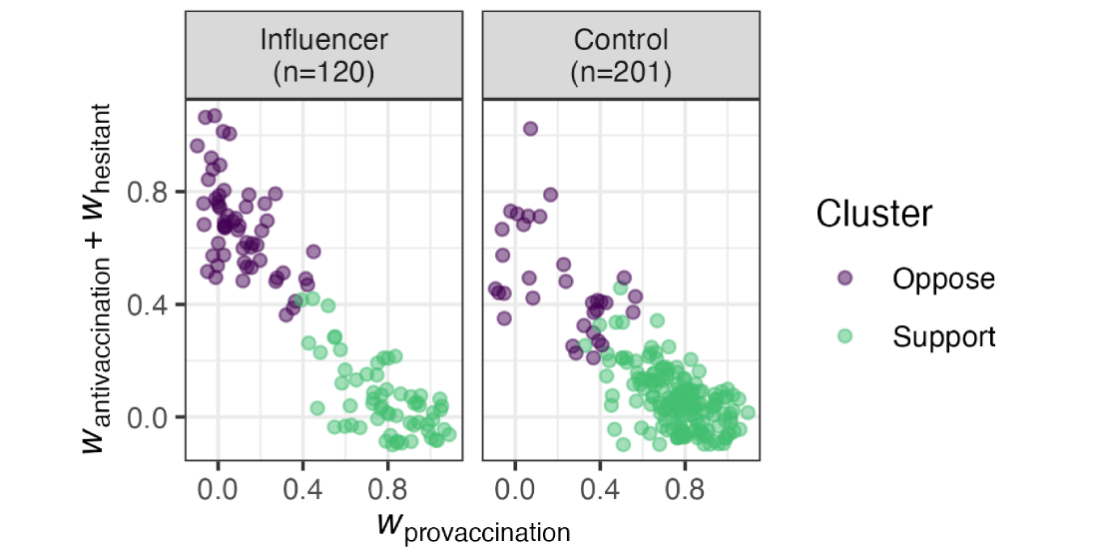
Result of k-means clustering of user stance vectors split by group. The x-axis is the provaccination component of the stance vector (defined in Equation 4). The y-axis is the sum of the antivaccination and hesitant components of the stance vector. Light jittering (+/- 0.1) has been added to reduce overplotting.

Inspecting the resulting clusters (Figure 3), 91 accounts were assigned to a predominantly antivaccination cluster, whereas 230 were assigned to a predominantly provaccination cluster. Within the influencer group, 58 out of 120 (48%) accounts were assigned to the antivaccination cluster. Within the control group, 33 out of 201 accounts (16%) were assigned to the antivaccination cluster. The difference in proportions was statistically significant (2-sample test for equality of proportions; *χ*^2^_1_[N=321]=36*.*1, *P<.*001). This modeling exercise suggests that while antivaccination stances were a minority opinion in both groups, they were relatively more common among influencer accounts.

### Topical Analysis of Argument Strategies for and Against Vaccination

#### Overview

To understand the rhetorical strategies used to promote or oppose vaccination, we once again used the BERTopic modeling library to cluster influencers’ tweets (more details in the Methods section). Tables 5 and 6 show the top 5 themes that emerged from topic modeling for both anti- and provaccination tweets by wellness influencers. Among tweets opposed to vaccination, the most prevalent theme was criticism of recommending COVID-19 vaccination for children. Other major themes included government overreach through vaccine mandates, corruption of the pharmaceutical industry (“big pharma”), opposition to recurring booster shots, and rejection of vaccine passports. Taken together, these topics reveal that wellness influencers who opposed COVID-19 vaccination often did so by sowing broader suspicions toward government and scientific institutions.

Among provaccination tweets, the largest cluster appears to represent encouragement and calls to action. Other major themes include broadcasting information about vaccine efficacy and safety, criticizing “antivaxxers,” and discussing immunity. In addition, 3 of the clusters (vaccine efficacy, vaccine safety, and immunity) appear to involve sharing scientific (or at least scientific-sounding) information. While it is beyond the scope of this study to fact-check all scientific appeals made in the tweets, it is notable that adopting a scientific framing is a popular rhetorical strategy.

**Table 5:** Antivaccination themes in wellness influencer tweets.

Cluster theme	Number of tweets, n	Example
Protecting children	56	“White house says it is time for the vulnerable 5-year-olds to roll up their sleeves and take the COVID vax.”
Government overreach	55	“Vaccines were supposed to be the way to freedom, they’re clearly the way to more authoritarianism.”
Big pharma	55	“CDC admits it: Big Pharma’s injured 387,087; seriously injured 31,240.”
Recurring booster shots	45	“If you want a picture of the future, imagine a booster injecting into a human arm, forever.”
Vaccine passports	45	“Just say no to vaccine passports.”

**Table 6:** Provaccination themes in wellness influencer tweets.

Cluster theme	Number of tweets, n	Example
Encouraging vaccination	108	“We all have to do our part and be vaccinated for the greater good.”
Vaccine efficacy	98	“The Covid Vaccine is 80% effective after 8 months of development, when the flu vaccine is 40% effective after 70 years.”
Vaccine safety	55	“Vaccines are absolutely safe. There is nothing to worry about. There is MUCH MORE risk from a Covid illness than from a vaccine.”
Criticizing vaccine skeptics	41	“The Wisconsin vaccine saboteur was a microchip guy and a flat Earther.”
Immunity	32	“We need 80-90% of us vaccinated to reach herd immunity and put covid in the rear view mirror.”

#### Temporal Dynamics in Vaccine Support

Finally, we investigated temporal dynamics in vaccine support: was support relatively consistent across time? Were there different temporal dynamics for the influencer and control groups? Note that these questions were developed post hoc, and as such, we had no hypotheses in mind about what we would observe. These findings should, therefore, be considered exploratory.

**Figure 4:**
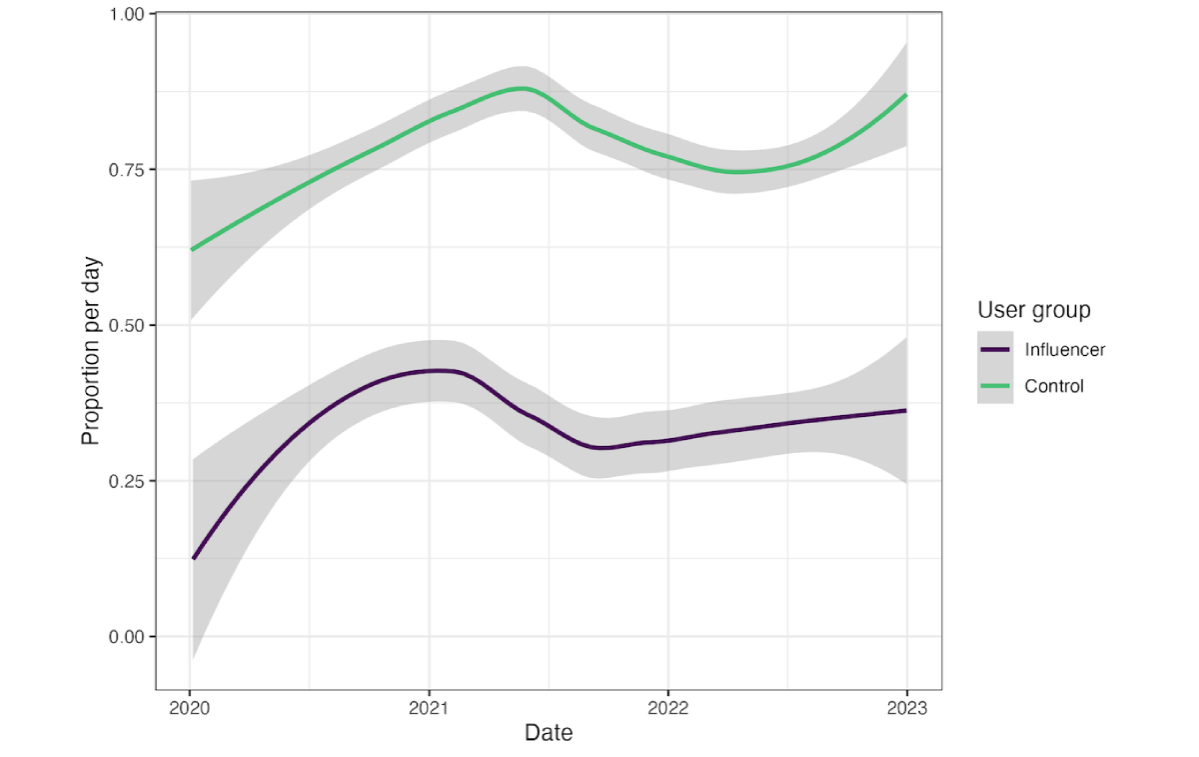
The proportion of provaccination tweets per day, out of all stance-taking tweets about vaccines, during the observation period. Loess smoothing has been applied for ease of interpretation, and shaded ranges indicate one standard error. Standard errors are wider at the start and end of the observation period when there were relatively fewer on-topic tweets per day.

Trends in daily support (as a percentage of stance-taking tweets) for the two groups are shown in Figure 4. We see that both groups followed a relatively parallel trajectory, with the key difference being the baseline level of support (ie, the trend curves are similar, except influencers are shifted lower on the y-axis compared to controls). The proportion of supportive messaging peaked in the first half of 2021 for both groups. It is challenging to directly align this peak with a clear indicator of vaccine access, as we are unable to verify the location of most users in our sample, and vaccine rollout schedules differ by country.

Despite this ambiguity, we can note a few milestones in global vaccine rollout: the first authorized COVID-19 vaccines after a large clinical trial became available in December 2020, starting in the United Kingdom and quickly followed by other nations [66] (though note that Russia and China had already begun distributing candidate vaccines based on intermediate clinical trial results). The number of COVID-19 vaccines administered per day around the world increased from December 2020 until it peaked around June 2021, with a smaller peak in daily vaccination counts around December 2021 [67].

If a large share of provaccination messaging from influencers related to encouraging people to get vaccinated (more details in Table 6), then it seems reasonable that this messaging would be concentrated while vaccine distribution was reaching its highest velocity—during the first half of 2021. Although trendlines for both groups appear to rise again at the end of 2022, we urge caution interpreting this—there are simply fewer on-topic tweets per day at the beginning and end of the observation period (more details in Figure 2), so estimates of the prevalence of provaccination stances will necessarily be noisier. This is visible in the wider standard error ranges in early 2020 and late 2022.

While it is possible that temporal dynamics in supportive messaging are related to accounts changing stances, it seems more likely that the burst of provaccination messaging reflects activity by a group of accounts that were consistently provaccine. This prediction is based largely on our user clustering analysis, in which the influencers group appears to have distinct stance clusters rather than a uniform continuum (see Figure 3). If many users were switching their opinions, we would expect a more continuous distribution, as averaging a mixture of pro- and antivaccination stances should lead to an intermediate valence.

However, further study would be required to definitively estimate the prevalence of stance changes. Detecting changes reliably would require statistical power beyond what we are able to achieve through a 10% random sample of tweets alone—at an absolute minimum, there would need to be at least two stance-taking tweets present per account at sufficiently separate time points (and in practice, the number will be even higher because the stance-detection model is not perfectly reliable). This question could ideally be answered using a more complete archive of social media activity.

## Discussion

### Summary of Findings

This study provides evidence that wellness influencers on Twitter were more likely to oppose COVID-19 vaccination compared with other Twitter users participating in health discussions before the pandemic. In our analysis, we identified a cohort of wellness influencer accounts during 2019, before the onset of the global coronavirus pandemic. Compared to a control group of accounts that posted on similar topics, wellness influencers were more likely to tweet messages expressing antivaccination stances during the rollout of the COVID-19 vaccine. Among wellness influencer accounts for which we could estimate vaccine stance, roughly half (48%) were identified as opposing vaccines, compared with 16% of accounts in the control group. This overall finding was robust to incorporating covariates into our statistical models, such as the social network centrality of an account or whether an account represents an individual or not.

In addition, we conducted an exploratory analysis of themes in pro- and antivaccination tweets by influencers. Our topic modeling approach provides further evidence that anti-establishment messaging comprises a core part of many wellness influencers’ rhetoric. These accounts invoked themes like parental rights, government overreach, and distrust of corrupt pharmaceutical companies when opposing vaccination. Provaccination influencers, on the other hand, encouraged followers to get vaccinated and shared scientifically framed information about vaccine safety, efficacy, and its relationship with immunity. Their use of scientific framing suggests these influencers recognize the cultural authority science holds.

### Implications

Our findings are congruent with the hypothesis raised by Baker and Rojek [24,25] that the rise of wellness influencers is a direct response to waning public trust in traditional authorities like medical professionals or public health agencies. Notably, the dominant antivaccine messaging themes among wellness influencers we observed were related to parental rights, government overreach, and corruption. While their tweets often contained scientific-sounding language, the rhetoric was markedly political.

If these influencers are indeed popular because of preexisting low trust in public institutions that make health knowledge and policies, then attempts to better regulate or counteract wellness content online may have limited impact. Indeed, a 2021 meta-analysis of COVID-19 misinformation mitigation strategies, such as offering corrections by experts or peers, found overall no statistically significant effect for these interventions online or offline [21]. This does not prove counter-messaging against vaccine misinformation never works, but suggests useful interventions are likely to be context-specific (ie, tailored to a particular community) with modest effect sizes.

Based on our results and the current literature, we would recommend a more direct—though ultimately far more challenging—approach: proactively investing in efforts to restore trust in public institutions to stem the tide of interest in wellness influencers before major public health crises occur.

### Limitations

#### Sources of Measurement Error

Our work has several limitations. The findings here are limited by reliance on Twitter data, particularly with a 10% random sample of tweets. Because we cannot query any user’s entire tweet history and the stance detection model is not perfectly accurate, our estimates of the prevalence of pro- and antivaccination messaging are all probabilistic. In addition, while some users produced a high volume of stance-taking tweets in our dataset, for others, we could only recover a single stance-taking tweet. Our group-level analyses should, therefore, be treated with higher confidence than our individual-level analyses, which are surely noisier.

#### Sample Size and Generalizability

Second, our sample size is relatively small for a social media study with only a few hundred Twitter accounts. Our criteria for labeling influencers are conservative, meaning they likely have a high false negative rate and a low false positive rate. In other words, while we are confident that the accounts we have identified as influencers meet our criteria, we have likely missed similar accounts that simply did not have as many tweets present in the Twitter Decahose random sample to be detected. Because we are attempting to make an argument about how a distinct subset of Twitter users behave, we prefer this conservative approach, which prioritizes the validity of our sample over a larger dataset. However, this design choice implies that our results should not be taken as a comprehensive view of support for vaccination on Twitter broadly.

#### Consequences of The Twitter API Change

When we first designed this study and carried out our initial exploratory work in the space, researchers had broad access to Twitter’s free API, which could be used to query information about accounts like their bio, locations, full tweet history, and followers. In February 2023, however, Twitter ended free access to its API, including for researchers. This blockage required us to rethink several aspects of our study, as it effectively limited us to using only what data could be accessed through the Twitter Decahose. The Decahose, while an extraordinary resource for researchers, stores only information about tweets rather than user accounts. Consequently, we can only learn about users through the tweets attached to their account handle in the random 10% sample.

The API closure required us to make several major changes to our research strategy. While we had initially intended to identify influencers through a network analysis using Twitter lists of wellness influencers as the seed, the API shutdown meant we could no longer easily assemble a complete follower network (or collection of lists). Instead, we reconstructed the social network through retweets in the Decahose dataset during 2019 and used keywords to ensure topical relevance.

In addition, we planned to use researcher tools such as Botometer [63] (to detect bots in our sample) and M3-Inference [68] (to label accounts corresponding to individuals and infer demographic variables) to programmatically identify candidate influencer accounts at scale. However, both these tools require Twitter API access, and as such, we were unable to incorporate them. This led us to use stricter criteria for selecting candidate influencer accounts from keyword usage to produce a smaller volume of candidate accounts amenable to manual review.

Furthermore, we had intended to pull the entire available tweet history for users identified as influencers or members of the control group through the API, which would have given us a better statistical estimate for vaccine stance per individual. Although we were still able to address our core research questions, better estimates of stance at the individual level would have allowed us to look at interactions with demographic variables, topical interests, and time (to measure the prevalence of stance-changing).

While we hope our work in its current form is still useful to the broader research community, our experience also testifies to the serious consequences of the API shutdown on social media research—both for data access and for the tremendous ecosystem of open-source software tools for researchers built around Twitter.

### Further Research Directions

While our study provides an initial estimate of the prevalence of antipublic health messaging in a cohort of wellness influencers, further research will be needed to fully understand the reach and impact of this messaging. The measures in our study focus on the content of tweets rather than their effect on readers. We do not know how many people saw the vaccine-related tweets in our sample or the demographics of the audience for the tweets.

Perhaps more importantly, we cannot decisively say whether the rhetoric used by wellness influencers was persuasive to followers. There has been recent attention on the “wellness to conspiracy” pipeline [26,69,70], which proposes that participation in online wellness communities can lead to exposure to—and even adoption of—conspiracy beliefs (for example, the American far-right conspiracy Q-Anon). While it is clear from the literature that some individuals have followed such a path, it remains possible that the pipeline is narrow: just as science denial is characterized by picking and choosing select aspects of scientific consensus to reject [71-73], people who follow wellness influencer accounts for diet suggestions might discount influencer’s opinions on vaccines.

One way to directly assess the downstream effects of following wellness influencers or participating in wellness communities online would be to follow users over time, an approach that has been used to study pathways toward and away from conspiracy beliefs on Reddit [74,75]. Considering that the researcher API for Twitter has disappeared indefinitely, further studies would likely have to be conducted on other social media platforms. Transitioning to another social media platform for research would also provide an opportunity to check that our main findings replicate in other online spaces.

An additional direction for research would be on protective factors observed in wellness influencers who go on to support public health initiatives. We observed that roughly half of the wellness influencers in our sample who expressed a stance on the COVID-19 vaccines voiced support. These users need to craft messaging that preserves their own authority as health experts of some sort while also expressing a position that broadly supports establishment sources of knowledge. Identifying their messaging strategies and potential motivations (both commercial and ideological) could illuminate productive avenues for public health leaders and social media personalities to work together.

## Abbreviations

API: application programming interface
HDBSCAN: hierarchical density-based spatial clustering of applications with noise
UMAP: Uniform Manifold Approximation and Projection
